# Human immunity to *Toxoplasma gondii*

**DOI:** 10.1371/journal.ppat.1008097

**Published:** 2019-12-12

**Authors:** Daniel Fisch, Barbara Clough, Eva-Maria Frickel

**Affiliations:** Host-*Toxoplasma* Interaction Laboratory, The Francis Crick Institute, London, United Kingdom; Duke University School of Medicine, UNITED STATES

## Innate immune response and *Toxoplasma* detection

*Toxoplasma gondii* (*Tg*) is an apicomplexan parasite able to invade any nucleated cell in warm-blooded animals. Approximately 30% of all humans harbour a chronic and asymptomatic infection [1]. Health risks include severe encephalitis in the immunocompromised, congenital defects, and ocular disease prevalent mostly in South America [2]. Felines are *Tgs* definitive host, and human infection occurs mostly through consumption of contaminated food or water [3]. Following ingestion, an initial site of infection develops in the tissue surrounding the intestines, causing inflammation. From there, *Tg* disseminates via the blood stream, establishing a chronic infection and entering immune-privileged sites, including the brain [4]. As humans are dead-end hosts, the interaction between host and parasite differs from that of rodents, and it is surprising how little is known about several levels of the human response to infection [5,6]. Hence, the use of mice as a model can only contribute partially to the study of the human response to *Tg*-infection. In this review, we explore data available on the human immune response to *Tg-*infection on a systemic level, in the response of individual cells, and to the complete control of infection. We also discuss *Tg* entering immune-privileged sites—causing disease in healthy individuals—and propose areas of interest for future research.

The innate immune system is the first to respond to infection with production of interleukin (IL)-12 by neutrophils, dendritic cells (DCs), and monocytes but not macrophages that have phagocytosed *Tg* [7–9]. Intracellular sensing differs from mice as humans do not have functional equivalents to murine toll-like receptors (TLRs)11 and 12 [10,11]. Monocytes sense *Tg*-infection through Alarmin S100A11 secreted from infected cells, which results in production of the chemokine (C-C motif) ligand 2 (CCL2) [12]. Additionally, cytosolic recognition of *Tg* in monocytes relies partly on the NLR family pyrin domain containing 1 (*NLRP1)* and *NLRP3* inflammasome, leading to cell death at later time points and early secretion of IL-1β [13,14]. Interestingly, neutrophils and macrophages do not sense *Tg*-infection in the same way since they do not display pyroptosis or IL-1β secretion [14,15].

**Fig 1:**
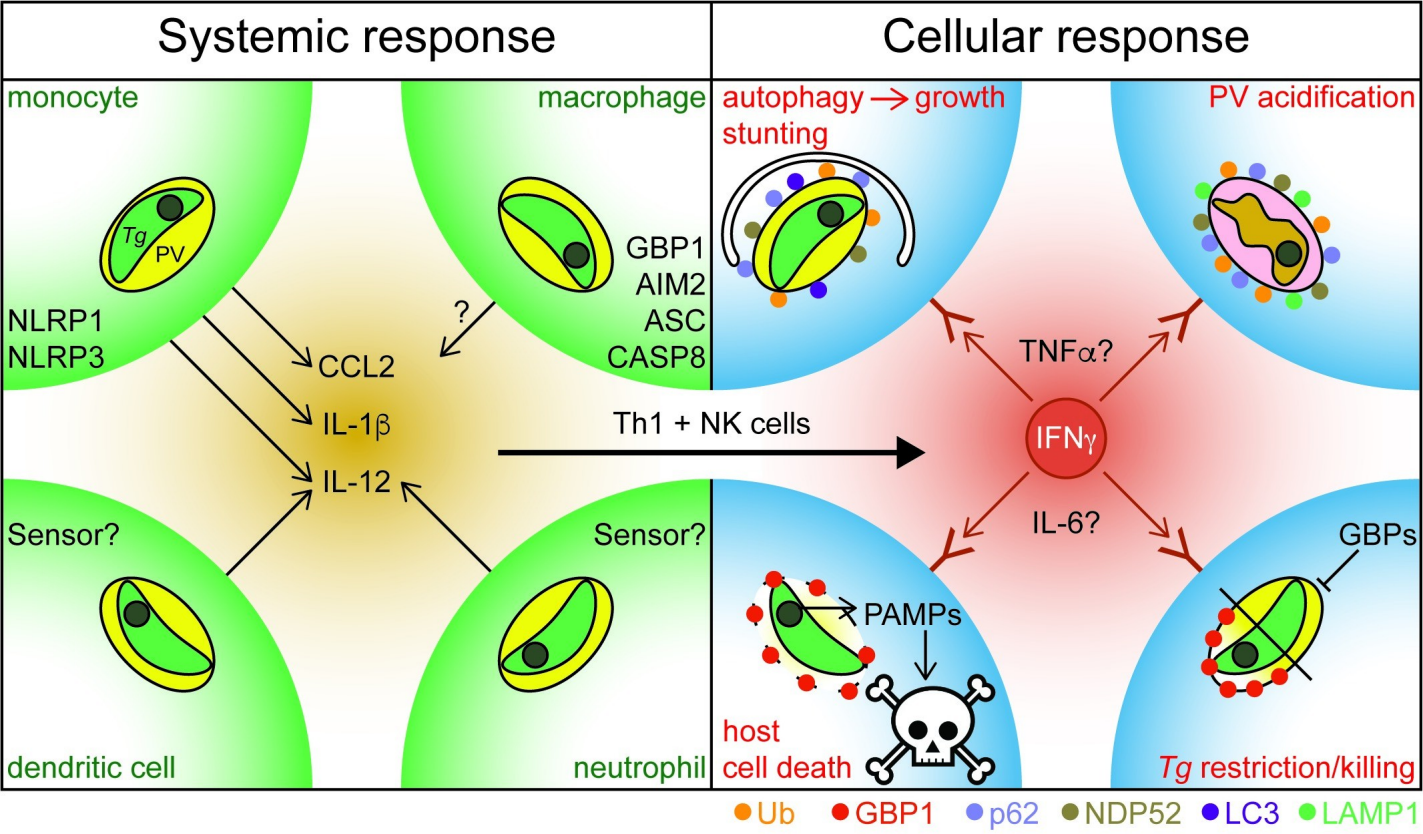
Systemic and cellular human response to *Toxoplasma* infection. Left: Systemic response to *Tg*-infection: Infected cells and cells that have phagocytosed *Tg* parasites at a site of infection sense the presence of the pathogen via the indicated PRRs and defence proteins and react by production of proinflammatory cytokines and chemokines like CCL2, IL-1β, and IL-12. This cytokine presence will trigger IFNγ–production by Th1 and NK cells. Right: Cellular response to *Tg*-infection: IFNγ and potentially other cytokines trigger infected cells to mount a cell-intrinsic defence against the PV. Mechanisms include ubiquitin-driven non-canonical autophagy of the entire PV and growth stunting; marking the PV with Ub, LAMP1, and the autophagy adapter proteins NDP52 and p62, followed by acidification of the vacuoles and killing of the parasite; recruitment of GBP1 to *Tg* vacuoles to disrupt them and expose the parasite within or growth restriction of *Tg* by GBP1 without translocation to the vacuole; and host cell death in response to opened PVs and leakage of pathogen-associated molecular patterns into the cytosol for detection by PRRs. The exact mechanisms highly depend on the cell type and the *Tg* strain infecting the cells. AIM, absent in melanoma 2; ASC, apoptosis-associated speck-like protein containing a CARD; CASP, caspase; CCL, chemokine (C-C motif) ligand; GBP, guanylate binding protein; IL, interleukin; IFNγ, interferon gamma; LAMP, lysosome-associated membrane protein; NDP52, nuclear domain 10 protein 52; NK, natural killer; NLRP, nucleotide-binding oligomerization domain, Leucine rich repeat and Pyrin domain containing; PAMP, pathogen-associated molecular patterns; PRR, pattern recognition receptor; PV, parasitophorous vacuole; *Tg*, *Toxoplasma gondii;* Th, T helper cell; TNFα, tumour necrosis factor α; Ub, Ubiquitin.

Based on *in vivo* mouse and human *in vitro* models, cytokine production in the inflamed tissue triggers interferon gamma (IFNγ)-production by T helper cell (Th)1 and natural killer (NK) cells, which leads to a robust adaptive Th1-immune response to control *Tg*-infection (see Fig 1) [16,17].

## Cellular response to *Toxoplasma* infection

The human cellular response to *Tg*-infection is highly dependent on cell type [18] and the infecting strain of *Tg* [19]. Interestingly, although the principal cytokine controlling *Tg*-infection is IFNγ, other cytokines have been implicated. For example, brain microglial cells control *Tg* growth by production of tumour necrosis factor α (TNFα) and IL-6 [20]. TNFα is proposed to mediate *Tg* killing in patients with IFNγ receptor 1 (*IFNGR1*) deficiency, partially compensating for lack of IFNγ-responsiveness [21]. Furthermore, IFNγ-independent control of *Tg* has been reported via cluster of differentiation (CD)40-induced autophagy of parasitophorous vacuoles (PV) in human macrophages [22], with the caveat that *Tg* activates epidermal growth factor receptor (EGFR) to combat its own autophagic clearance [23]. It is likely that several different host response pathways act in concert to control *Tg*-infection. However, for the purposes of this review, we will focus on the role of IFNγ-induced defence mechanisms (see Fig 1).

We recently showed that the IFNγ dose-dependent restriction of *Tg*-infection depends on cell type, with epithelial cells displaying a sharp parasiticidal effect, in contrast to macrophages, fibroblasts, and endothelial cells demonstrating a dose-dependent response [24]. A common initial response is to mark PVs with ubiquitin [24–26], but the ubiquitinated substrate(s) and the E3 ligases involved in this process remain to be defined. Following ubiquitination, epithelial cells control parasites through an incomplete autophagy, involving recruitment of autophagy adapter proteins p62 (*SQSTM1*) and NDP52 (*CALCOCO2*) and the autophagy-related protein 8 (Atg8) protein microtubule-associated protein-1A/1B light chain 3 (LC3B), but this fails to mature to autophago-lysosomes [25]. Endothelial cells follow this path up to the recruitment of adapter proteins, but then deviate from it, either by shuttling the marked PV into the endo-lysosomal pathway or directly acidifying the PVs [26]. Both cell types maintain the integrity of the PV and restrict the parasite within.

Conversely, macrophages are able to open *Tg* vacuoles, as we have recently demonstrated [27]. This induces an atypical apoptosis pathway relying on DNA-sensing by absent in melanoma 2 (*AIM2*) and execution of apoptosis via an apoptosis-associated speck-like protein containing a CARD (ASC)-caspase 8 (*CASP8)* signalling axis [29], whereas *Tg* is able to block apoptosis in other human cells [28]. This cell death phenotype is dependent on IFNγ-induced guanylate binding proteins (GBPs), of which GBP1 translocates to *Tg* vacuoles and releases *Tg*-derived molecular ligands of cellular receptors [27]. Similarly, GBP1 translocates to *Tg* vacuoles in mesenchymal stem cells [29] but not in epithelial cells [30]. In both cell types, the protein was able to restrict *Tg* independent of its recruitment [29,30]. Thus, recruited GBP1 seems to uniquely induce host cell death in macrophages. In contrast to death of macrophages, IFNγ-primed fibroblasts die through an uncharacterised form of cell death [31].

Similar to GBP1’s function in restricting *Tg* growth remotely from the PV, other cell-intrinsic mechanisms act on *Tg* from a distance: IFNγ-induced indoleamine-2,3-dioxygenase 1 (IDO) can deplete cells of tryptophan, which slows down growth of tryptophan-auxotrophic *Tg* [32]. This mechanism can be counteracted by the *Tg* effector protein inhibitor of STAT1 transcriptional activity (*Tg*IST) [33] and has been shown to be dispensable in human umbilical vein endothelial cells (HUVECs) [34]. In general, the secreted *Tg* virulence factor *Tg*IST is able to shut down many IFNγ-mediated responses to infection by blocking transcription of IFNγ-induced genes [35,36]. Furthermore, in contrast to mouse cells, nitric oxide was not relevant in restriction of *Tg* in HUVECs [34].

Parasites that escape within a few hours of entering the host cells may be an important unexplored consequence of IFNγ-dependent host restriction, as has been observed in human fibroblasts and endothelial cells [31]. This phenomenon is difficult to quantitate and may be larger than the 5%–10% reported. Whether the escaped *Tg* parasites are viable remains an open question.

Taken together, cell-intrinsic defence to *Tg*-infection not only differs largely between species but also between different cell types, and much work is needed to uncover new mechanisms.

## Dissemination in the infected host and entering of immune-privileged sites

While the host responds to *Tg*-infection with cell death and cytokine production in the infected tissue, some parasites leave the site of primary infection and disseminate in the body [4]. Under pressure from the adaptive immune response, *Tg* converts to the bradyzoite stage and forms tissue cysts, surviving until death of the host [37]. *Tg* is believed to travel to immune-privileged sites of the brain and eye and also cross the placenta of an infected woman and infect the foetus congenitally [4]. In primary infections, this can result in abortion or foetal abnormalities such as hydrocephalus and retinochoroiditis [2,3].

Retinochoroiditis is the most common form of congenital toxoplasmosis, with the infection leading to an increase in HIF1α and vascular endothelial growth factor (VEGF) expression, resulting in increased vascularisation [38]. An increase in IL-1β, IL-6, granulocyte-macrophagecolony-stimulating factor (GM-CSF), and intercellular adhesion molecule (ICAM)-1 produced by retinal pigment epithelial cells was also described [39]. Intraocular fluid of *Tg*-infected eyes contain elevated levels of TGF-β [40,41], which may modulate the effects of IFNγ that inhibit *Tg* replication in human primary retinal pigment epithelial cells by tryptophan starvation [42]. Differences between French and Colombian clinical cases of ocular toxoplasmosis highlight the importance of understanding disease severity. Decreased intraocular IFNγ and IL-17, and higher IL-13 and IL-6 expression were detected in Colombian patients [43], suggesting the increased severity of ocular disease caused by South American strains could be attributed to an inhibition of protection afforded by IFNγ.

## Concluding remarks and future studies

The immune response to *Tg* has been extensively studied in mice. Since mice are an intermediate host to *Tg*, many mechanisms are unique to this host–pathogen pair and cannot be extrapolated to the human host. Key areas of research for the future are as follows:

1. *IFNGR1*-deficient patients possibly do not suffer from a higher incidence of *Tg*-borne disease. Is IFNγ the main cytokine responsible for *Tg* control in humans in all cell types?
2. South American atypical *Tg* strains cause ocular toxoplasmosis, but other strains do not cause this disease. What makes these strains unique in being a disease-causing pathogen?
3. Virulence of the clonal *Tg* strains was defined based on the mouse system and is not transferable to the human host. What are *Tg* virulence factors in humans and which strains are they derived from?
4. Different human cell types show varying responses to *Tg*-infection. Why is there no unified defence strategy?
5. Recruitment of host effectors to vacuole is only 30%–50% at any one time point. Which host defence mechanisms operate away from the vacuole?
6. How many *Tg* tachyzoites escape acute phase control? What host response triggers parasites to escape the cell and are they still viable?

These and other open points will have to be addressed in future studies to uncover new mechanisms of the human response to *Tg*-infection.
